# Nanocomposites with Optimized Polytetrafluoroethylene Content as a Reinforcement Agent in PA12 and PLA for Material Extrusion Additive Manufacturing

**DOI:** 10.3390/polym15132786

**Published:** 2023-06-22

**Authors:** Nectarios Vidakis, Markos Petousis, Amalia Moutsopoulou, Vassilis Papadakis, Mariza Spiridaki, Nikolaos Mountakis, Chrysa Charou, Dimitris Tsikritzis, Emmanuel Maravelakis

**Affiliations:** 1 Department of Mechanical Engineering, Hellenic Mediterranean University, 71410 Heraklion, Greece; markospetousis@hmu.gr (M.P.); amalia@hmu.gr (A.M.); tm20084@edu.hmu.gr (M.S.); mountakis@hmu.gr (N.M.); charou@hmu.gr (C.C.); 2 Department of Industrial Design and Production Engineering, University of West Attica, 12244 Athens, Greece; v.papadakis@uniwa.gr; 3 Institute of Electronic Structure and Laser, Foundation for Research and Technology–Hellas, N. Plastira 100m, 70013 Heraklion, Greece; 4 Department of Electrical & Computer Engineering, Hellenic Mediterranean University, 71410 Heraklion, Greece; dtsikritzis@hmu.gr; 5 Department of Electronic Engineering, Hellenic Mediterranean University (HMU), 73133 Chania, Greece; marvel@hmu.gr

**Keywords:** polyamide 12 (PA12), polylactic acid (PLA), polytetrafluoroethylene (PTFE), additive manufacturing technology (AM), material extrusion (MEX), nanocomposites, thermomechanical characterization

## Abstract

Herein, polytetrafluoroethylene (PTFE) is evaluated as a reinforcement agent in material extrusion (MEX) additive manufacturing (AM), aiming to develop nanocomposites with enhanced mechanical performance. Loadings up to 4.0 wt.% were introduced as fillers of polylactic acid (PLA) and polyamide 12 (PA12) matrices. Filaments for MEX AM were prepared to produce corresponding 3D-printed samples. For the thorough characterization of the nanocomposites, a series of standardized mechanical tests were followed, along with AFM, TGA, Raman spectroscopy, EDS, and SEM analyses. The results showed an improved mechanical response for filler concentrations between 2.0 and 3.0 wt.%. The enhancement for the PLA/PTFE 2.0 wt.% in the tensile strength reached 21.1% and the modulus of elasticity 25.5%; for the PA12/PTFE 3.0 wt.%, 34.1%, and 41.7%, respectively. For PLA/PTFE 2.0 wt.%, the enhancement in the flexural strength reached 57.6% and the modulus of elasticity 25.5%; for the PA12/PTFE 3.0 wt.%, 14.7%, and 17.2%, respectively. This research enables the ability to deploy PTFE as a reinforcement agent in the PA12 and PLA thermoplastic engineering polymers in the MEX AM process, expanding the potential applications.

## 1. Introduction

Additive manufacturing technology (AM) is a method deployed for turning a variety of materials into construction parts [1]. The seven additive manufacturing technologies are vat polymerization, the sheet’s lamination, the material’s extrusion (MEX), the material’s jetting, the binder’s jetting direct energy deposition, and the powder’s bed fusion. They are utilized for prototyping and manufacturing purposes. They can fabricate light and stable highly complex geometries without using any tool [2]. Three-dimensional printing (3DP) is classified in the category of an AM method, which contributes to constructing 3D parts made out of composites arising from different materials such as metals, polymers, and ceramics, i.e., thermosets, thermoplastics or elastomers [1].

FFF (fused filament fabrication) is a MEX 3D printing process that can be found in a great range of applications in the manufacturing sectors, such as (i) biomedical, (ii) aerospace, (iii) automobile, (iv) pharmaceutical, (v) construction, (vi) electrical and electronics, (vii) food, (viii) textile, (ix) jewelry, (x) toys, (xi) sports, (xii) energy, etc. [3]. According to that method, a thermoplastic polymeric filament is heated. The extrusion head is electronically led to the desired paths, fabricating the requested geometries using computer control [1]. Some of the 3D printing parameters the FFF technique has are the percentage of the infill, the pattern of infill, the number of contours, the raster angle and style, the thickness of the layers, the speed of the printing process, the temperature of printing, the temperature of the bed, the air gap and the number of solid laminates (top/bottom) [3].

The biodegradable and biocompatible properties of polylactic acid (PLA) as a polyester thermoplastic [4] have led to an extended investigation for nanocomposite applications [5]. Due to its biocompatibility, nanocomposites have been developed with antimicrobial properties to satisfy the specifications of medical and culinary applications [5]. PLA contains a distinctive mechanical strength, toughness, impact, resistance, and ease of melt-processing, which justifies its wide usage as feedstock material in FFF 3DP technology [6]. As expected, in MEX 3D printing, the response of PLA has been thoroughly reported [4,7]. It can be renewable [8] when implementing the appropriate processing conditions of thermoplastic polyester and is utilized for packaging [9], agricultural products, disposable materials, biomedicine [8,10], membranes [11] as well as automotive applications [6]. The melting point (Tm) of PLA is considered to be high in the vicinity of ~150–160 °C and renders a suitable material not only for various biomedical uses but also for engineering applications [4,12].

Polyamide 12 (PA12) belongs to the category of polyamides, distinctive for their strength, toughness, and resistance to fracture and impact in cases where it is subjected to a significant amount of deformation [13,14]. Polyamide is usually utilized when it comes to parts production for the automotive [15] and aviation industries [16], marine applications [17], membranes [18] and filters [19], and biomedical applications [20] while it has been exploited in MEX 3D printing [21]. Its response in various types of applications has been reported, for example in 3D-printed thin wall structures [22]. It is thought to be a solid and durable material with a great melting point and minimal coefficient of friction, which is the reason it is a suitable material for functional printing gears, while it is also known for its hygroscopicity [23]. As shown, it is a popular engineering thermoplastic with its use expanded in the AM processes, in vat photopolymerization [24], and powder bed fusion [25]. In MEX 3D printing, its performance has been investigated [21]. Additionally, nanocompounds with antibacterial capabilities for medical applications [20,26] have been reported. Polyamides have been exploited in hybrid AM technologies [27] to further expand their potential in industrial applications.

Polytetrafluoroethylene (PTFE) belongs to the category of fluoropolymers [28], which are thermoplastics and was chosen in nanoparticle (NP) form (nanopowder) as an additive for the purposes of this study. It can be used for the aim of coatings, thermal sealing, insulation, lubrication, and bearings as well as clinical applications [28]. Some of its properties are, amongst others, mechanical strength, high thermal conductivity (when in composite form), chemical inertness, and hydrophobicity [29]. PTFE has a melting point of an average of 325 to 335 °C, which justifies the reason why it belongs to the thermoplastics characterized by high values of thermal resistance, as well as a high operating temperature [28]. It is considered an extreme polymer [30] and due to its high wear resistance [31], it is used in membranes [32] and applications with extreme tribological requirements [33,34]. In the medical field, its unique properties make it suitable for stents and grafts [35], implant tubes [36], and medical device applications [37]. Three-dimensional printing has also been applied for the development of parts for the aforementioned applications, but it focused mainly on microstructures [38], needles [39,40], nanogenerators [41,42], hydrophobic surfaces, and devices [43]. To date, the usage of PTFE NPs as an additive for maintaining the mechanical improvement of 3D-printed objects composed of PLA and PA12 thermoplastic polymers, as well as the thermomechanical and fracture qualities of the 3DP samples, have not yet been studied. In the MEX 3D printing process, according to the authors’ best knowledge and the conducted literature review herein, research is still very limited.

In this study, for the first time, PTFE has been integrated as filler into PLA and PA12 thermoplastic polymers in order to produce 3DP nanocomposite specimens with improved mechanical performance compared to pure polymeric matrices. Both polymers are very popular in various applications as presented above. PA12 is a commonly used thermoplastic, while PLA is the most used thermoplastic in MEX 3D printing. Both are biocompatible (PA12 is medical-grade and PLA is biocompatible by itself). Therefore, both polymeric matrices have been investigated and applied in different applications in the medical field. On the other hand, PTFE has unique properties and in the medical field, it is used in advanced applications, as presented above. Combining the advanced properties of the produced nanocomposites with the advantages of MEX 3D printing has merit for advanced medical applications, further expanding the MEX 3D printing potential applications. Herein, there has been a comparison of the reinforcement mechanism for each of the polymeric matrices. The additive loading remained persistent at 1, 2, 3, and 4 wt.% in both the PA12 and PLA, with the aim of understanding the straightforward procedure, structure, and property relationship for PLA and PA12 PTFE nanocompounds. A thermomechanical process was followed, and the produced 3D-printed samples were characterized through various experimental procedures corresponding to international standards. The goal was to prepare nanocompounds with improved performance, through a nonchemical process, which can be easily industrialized, and to evaluate the performance of PTFE toward this result.

## 2. Materials and Methods

### 2.1. Raw Materials for the Fabrication of the Nanocompounds

This study was conducted using PA12 purchased by Arkema SA (Rilsamid PA12 AESNOTL, from Colombes, France) in the form of grains. The MVR of PA12 is 8.0 cm^3^/10.0 min at 235 °C/5 kg, the VST is 142 °C (following ISO 306/B50), the Tm is 180 °C (following ISO 11357-3) and the density is 1.01 g/cm^2^ (following ISO 1183), taking into consideration the specifications of the supplier.

PLA, having the composition of a rough powder, was purchased from Plastika Kritis S.A (from Heraklion in Crete, Greece), in 3052D grade and 116.000 g/mol as the molecular weight. In accordance with the technical data sheet supplied by their suppliers, the PA12 and PLA polymer grades that were used as the matrices for constructing the nanocompounds were suitable for the procedures of melt mixing, while no other additives, such as heat stabilization, lubrication, or UV stabilizer, were introduced in the prepared nanocompounds.

PTFE nanopowder (polytetrafluoroethylene (C2F4)n) was purchased from Nanografi Nanotechnology (ODTÜ Teknokent İkizler Binası B-1/H, Ankara, Turkey) in the form of a white nanopowder and with a molecular weight of 100.02 g/mol. Considering the material safety data sheet, the melting point ranges from 279 to 326 °C, the softening point in excess reaches over 320 °C and the thermal conductivity is 0.250 w (m*K). The surface area is 4.57 m^2^/g, the purity is 99.9% and their particle size is 260 nm.

### 2.2. Fabrication of Filaments, SEM and EDS Analyses of PTFE Powder, 3D Printing Process of PLA and PA12 PTFE Nanocomposites

The process followed in this work started with preparing the nanocomposites by adding the desired filler loadings. Separate mixtures were prepared for each nanocompound. The prepared nanocompounds were tested and, until the samples’ mechanical performance began to deteriorate, the loadings were increased. The samples were manufactured and subjected to experimental tests under the same experimental parameters in order for the results to be able to be compared and evaluated. PTFE additive was investigated with regard to its reinforcement effect when combined individually with the two aforementioned thermoplastics.

PA12 and PLA thermoplastic materials in a raw powder form were mixed with the PTFE filler at 1.0, 2.0, 3.0, and 4.0 wt.% concentrations in separate bowls. The PA12 blends that arose were dried at a temperature of 80 °C and the corresponding PLA blends at 50 °C for about eight (8) hours each before the filament extrusion process. A main concern when preparing nanocomposites is uniformly dispersing the additives [44] in the matrix, which is not an easy task to achieve [45]. A two-step process was implemented to reach the best possible dispersion of the PTFE as an additive in the matrix materials. It started with the first mixing of the raw materials using an extruder from Noztek (Shoreham, UK). Then, pellets were made by shredding the filaments using a shredder from 3devo (Utrecht, The Netherlands). The pellets that resulted from the shredding procedure were used in a second extrusion with the aim of producing a 3DP filament that would be suitable for the FFF technique. In this second extrusion that followed, a 3D Evo composer (Utrecht, The Netherlands) was utilized with a screw with a unique geometry for material mixing.

In the beginning, some tests were conducted to define the optimal filament extrusion parameters such as the speed of mixing, as well as the extrusion temperatures. The 3DEvo composer 450 (Utrecht, The Netherlands) used in this study consisted of four (4) different temperature zones in its chamber. The diameter of the 3DP filaments was continuously measured throughout the extrusion process through an optical device in the 3DEvo composer 450 (Utrecht, The Netherlands), with the acceptable range fluctuating at 1.68 mm ± 0.07 mm. The outcome of these preliminary tests was then utilized to secure a high-quality filament with minimum defects and the right diameter across its length. The extruder nozzle was followed by an air duct of the machine, which was responsible for the filament’s cooling procedure, affecting its roundness. After the filaments were produced, they were dried at 80 °C when it came to PA12 and 50 °C when it came to the PLA filament. The extrusion settings are presented in Table 1 below.

The FFF 3DP procedure was carried out by deploying a 3D printer (by Intamsys Co., Ltd., Funmat HT 3D FFF technology, from Shanghai, China). There were trials utilizing both pure and nanocomposite filaments for the PA12 and PLA polymeric matrices, whilst the urge to define the finest possible 3DP parameters for high-quality specimen manufacturing. All specimens were 3DP with 100% infill density, 40.0 mm/s of printing speed, and 0.2 mm layer height. The nozzle’s temperature was set at 270 °C and the bed temperature at 90 °C for the PA12 and PA12/PTFE nanocomposites, while the corresponding values for the PLA and PLA/PTFE nanocomposites were, respectively, 210 °C and 50 °C. It should be noted that the 3D-printed samples were manufactured with a linear infill pattern. The strand orientation was altered between +45 deg and −45 deg in the successive layers. This 3D printing structure reduces the anisotropy in the 3D-printed parts due to the more homogeneous structure. The parametric investigation of the effect of the printing direction on the mechanical performance of the 3D-printed parts was not within the scope of the study, which focused mainly on the effect of the WC NPs on the two polymeric matrices.

**Figure 1:**
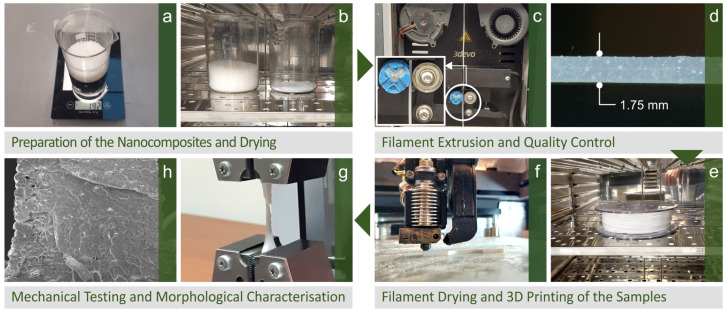
Presentation of the methodology followed while creating and testing the 3DP filament and specimens: (**a**) weighing and (**b**) drying the nanocomposite materials, (**c**) filament extrusion and (**d**) quality control, (**e**) drying of filament, (**f**) 3D printing of specimens, (**g**) testing specimens’ mechanical properties, (**h**) specimens’ morphological characterization (scanning electron microscopy—SEM).

The sequence of the procedure developed and implemented in this particular research to create the various specimens is schematically shown in Figure 1. These steps include starting with raw materials, preparing 3DP filaments, creating the various specimens, and then performing thermomechanical and morphological analysis.

**Figure 2:**
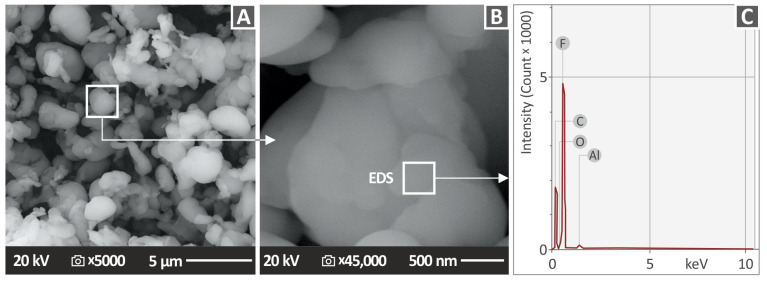
SEM analysis images of PTFE material in powder form at (**A**) ×5000 and (**B**) ×45,000 magnification, (**C**) EDS spectrum of PTFE powder.

The PTFE powder was initially inspected for its morphological and compositional characteristics through SEM and EDS analyses on a Jeol field emission apparatus (model name JSM IT700HR from Jeol Ltd., Tokyo, Japan). Figure 2 is a depiction of the PTFE SEM results on the PTFE nanopowder. Two different magnifications (Figure 2A,B) were used, with Figure 2B being a magnification of the area indicated in Figure 2A. The NPs’ shape was as per the manufacturer’s specifications. Their shape was round and random (the supplier does not disclose details regarding the NPs’ shape). Figure 2C shows the EDS spectrum. The EDS, though, is not suitable for precisely evaluating the stoichiometric ratio and the concentration of an element, considering the fact that the measurements were taken out of a small region. The high levels of F can be discerned between those of the rest of the elements by observing the graph. This confirms the presence of element F in the nanofiller (Figure 2C), which is logical considering the fact that PTFE has a chemical formula that contains fluorine (F) atoms [46]. The high peaks signify the existence of the elements’ high concentration in the observation region without the exact value of the quantities being specified by the EDS method. EDS was performed in the region indicated in Figure 2B, which by extent is a magnification of the region indicated in Figure 2A.

**Figure 3:**
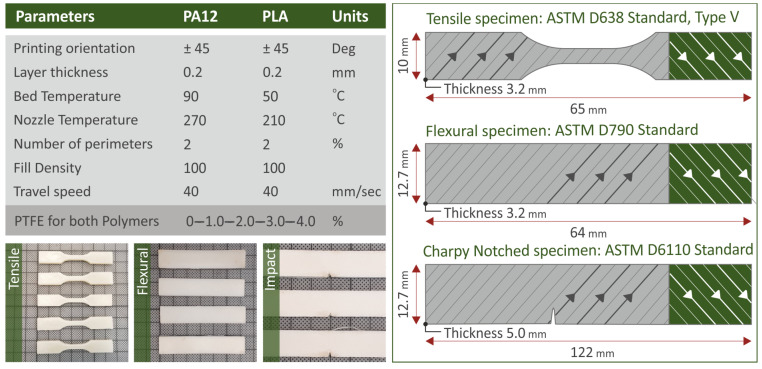
PA12 FFF three-dimensional printing parameters, PLA and PTFE nanocomposites, actual 3DP specimens, and representative samples of tensile, flexural, and Charpy notched specimens.

The geometry of the 3D-printed specimens utilized for the various mechanical characterizations, the optimal 3DP settings to fabricate the specimens, and the actual 3DP-made specimens in a representative sample form for tensile, flexural, and impact tests, are all shown in Figure 3. The parameters mentioned are namely the 3D printing orientation, layer thickness, bed temperature, nozzle temperature, number of perimeters, fill density, and travel speed, all referring to the PTFE nanocompounds for both of the polymers. These settings were determined with experiments and were also acquired from the literature [5,47].

### 2.3. Characterization Methods

A Raman spectrometer of type LabRAM-HR (HORIBA Scientific, from Kyoto, Japan) was utilized for Raman experiments, along with a laser beam with a 532 nm excitation level and 90 mW maximum output power. The stimulation light was supplied via a 50× focal length lens with an approximate aperture of 0.50 and a distance of operation of 10.60 mm (from LMPlanFL-N, Olympus, Tokyo, Japan) in order to detect the Raman activation.

All PA12, PA12/PTFE, PLA, and PLA/PTFE nanocomposites went through thermogravimetric analysis (TGA) while in a nitrogen atmosphere by engaging a TG/TDA Perkin Elmer Diamond (from Waltham, USA) device with a 10 °C/min step. Measurements were taken in the range of 30 to 550 °C.

A PPP-NCHR tip provided by Nanosensors (NanoWorld AG, Neuchâtel, Switzerland) (Si-doped tip, resonance frequency = 330.0 kHz, force constant 42.0 N/m) delivered the tapping mode AFM under ambient conditions. The second flattening process was followed by calculating the values of all filaments’ surface roughness utilized for 3DP from the total captured area of 10.0 × 10.0 μm^2^ of the corresponding samples’ height photographs. Then, 256 × 256-pixel images were captured, resulting in a lateral resolution of 39.1 nm. About 1.0 nm is the vertical resolution.

In the tensile investigations on the 3DP samples, a Jeol JSM IT700HR field emission SEM (Jeol Ltd., from Tokyo, Japan) was used in a vacuum setting at a 20 kV acceleration to conduct SEM characterization on the microstructure of both the cracked and the lateral surface of the samples (random tensile test samples were inspected). The observation of the samples took place with a secondary electron (SE) sensor. The samples were covered on a 5.0-nm-thin Au film in order to prevent charging impacts.

All the mechanical examinations were executed at a temperature of 23 °C. The tensile experiments corresponded with the guidelines of the ASTM D638-02a standard and the flexural experiments followed the ASTM D790-10 requirements (the spectrum of support was adjusted at 52 mm). An Imada MX-2 (produced by Imada Inc., from Northbrook, IL, USA) with an appropriate setup for tension mode and flexural tests was used. A testing speed of 10.0 mm/min was set in accordance with the corresponding standards. The impact tests corresponded with the ASTM D6110-04 guidelines. The tests were conducted with the use of a Terco MT 220 (provided by Terco, from Huddinge, Sweden) Charpy’s impact machine (releasing height of 367 mm, notched samples). The impact, tensile, and flexural test samples were all fabricated under identical 3DP settings. For each mechanical test, six (6) specimens from each nanocompound were evaluated.

Microhardness measurements followed the ASTM E384-17 requirements. An Innova Test-300 Vickers apparatus (produced by Innovatest Europe BV, from Maastricht, The Netherlands) was employed to assess the Vickers microhardness of the material. Each indentation lasted for 10 s and was made with an applied load of 100 gF. Each sample, which included raw PA12, pure PLA, and pure PLA/PTFE nanocompounds, had a total of six (6) indentations.

## 3. Results

### 3.1. Thermogravimetric Analysis

**Figure 4:**
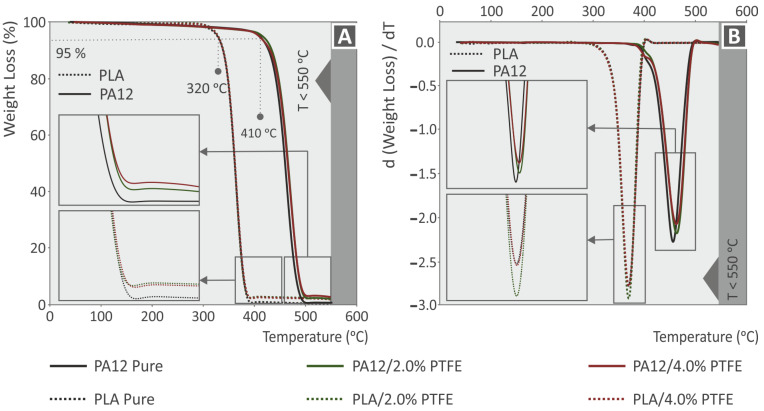
TGA (**A**) and DTG (**B**) analysis of 3DP neat PA12, PLA, and the corresponding 2 wt.%–4 wt.% PTFE nanocompounds.

In order to assess the temperature stability that characterizes each different material, TGA investigations have been conducted in this report in an N_2_ atmosphere [48]. The TGA graphs also helped to specify not only the nature but also the presence of the particular filler loading amount of each nanocomposite sample by observing the remaining material when the polymer matrix’s PA12 and PLA temperatures reached a level that caused their total decomposition. Figure 4 is a representation of the TGA and derivative thermogravimetry (DTG) (Figure 4A,B, respectively) curves of the 3DP PA12, PLA, PA12/2.0% PTFE, PA12/4.0% PTFE, PLA/2.0% PTFE and PLA/4.0% PTFE. By the graph outcome, PA12 is considered to be more thermally stable than PLA. Their decomposition temperature was found to be $T_{on}^{d}$ = 410 °C for PA12 and $T_{on}^{d}$ = 320 °C for PLA. It can also be observed that both of the polymeric materials have fully decomposed when the temperature level is above 500 °C, while the remaining material corresponds to the PTFE nanofiller concentration in each nanocompound.

By observing the TGA and DTG curves, it can be concluded that the utilization of the PTFE as a filler can cause a marginal increase in both PA12 and PLAs thermal stability, as the $T_{on}^{d}$ has reached some slightly higher temperatures in the nanocomposites. While observing the DTG graphs, a subtle difference in the PLA with PTFE nanoparticles can be discerned when referring to the slightly lower weight loss to temperature ratio. On the other end of the spectrum, when adding PTFE to PA12. the temperature remains the same while decreasing the weight loss ratio. The TGA and DTG analysis showed the temperatures set for the conduction of 3DP filament melt-mixing and extrusion procedures, and the FFF 3DP manufacturing did not reach the virgin polymeric PLA and PA12 matrices’ decomposition temperatures; thus, the results provided herein were not affected by such phenomena.

### 3.2. Raman and EDS Analysis

**Figure 5:**
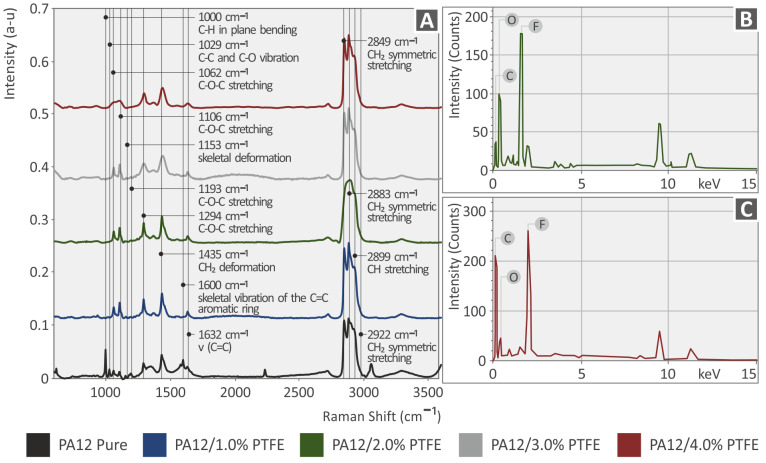
(**A**) Raman spectra of PA12 and PA12/PTFE at 1.0, 2.0, 3.0, 4.0 wt.% filler loadings nanocomposites, (**B**) EDS spectra of PA12/2.0% PTFE and (**C**) EDS spectra of PA12/4.0% PTFE.

**Figure 6:**
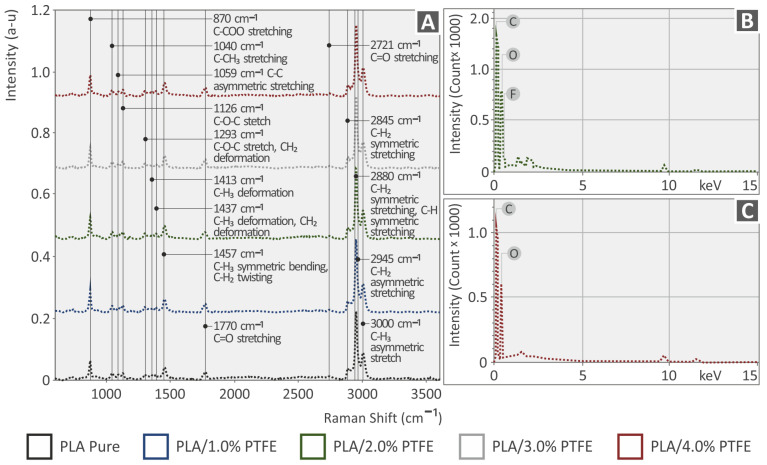
(**A**) Raman spectra of PLA and PLA/PTFE at 1.0, 2.0, 3.0, 4.0 wt.% filler loadings nanocomposites, (**B**) EDS spectra of 3DP PA12/2.0% PTFE and (**C**) EDS spectra of 3DP PA12/4.0% PTFE.

In Figure 5A, the clear Raman spectra from the pure material PA12, PA12 with 1 wt.% PTFE, PA12 with 2 wt.% PTFE, PA12 with 3 wt.% PTFE and PA12 with 4 wt.% PTFE, and in Figure 6, the corresponding spectra for the pure material PLA, PLA with 1 wt.% PTFE, PLA with 2 wt.% PTFE, PLA with 3 wt.% PTFE and PLA with 4 wt.% PTFE is depicted.

The related Raman peaks from the PA12 and PLA pure samples are presented in the following Table 2 and Table 3, respectively, and are validated by the literature.

With the PTFE addition in PA12, the Raman lines presented no clear differences with changes in the noise level. Nonsignificant differences were identified with a decreased intensity at the wavenumbers: 1063 cm^−1^ (C-C asymmetric stretching), 1110 cm^−1^ (C-O-C stretching), 1294 cm^−1^ (C-O-C stretching), and 1636 cm^−1^ (C=C stretching).

In PLA samples, the addition of PTFE presented significant differences with increased intensity in Raman lines: 870 cm^−1^ (C-COO stretching), 1451 cm^−1^ (C-H_3_ symmetric bending/C-H_2_ twisting), 1770 cm^−1^ (C=O stretching), 2945 cm^−1^ (C-H_2_ asymmetric stretching) and 3000 cm^−1^ (C-H_3_ asymmetric stretch).

Figure 5B,C and Figure 6B,C present the results of the EDS analysis conducted on the 3D-printed PA12/PTFE and PLA/PTFE nanocompounds. Figure 5B displays the existence of O, C, and F in the PA12/2.0% PTFE nanocomposites, while Figure 5C displays the existence of O, C, and F in the PA12/4.0% PTFE nanocomposites. In Figure 6B, the existence of the elements C, O, and F in the PLA/2.0% PTFE nanocomposites can be seen, while Figure 6C depicts the presence of C and O in the PLA/4.0% PTFE nanocomposites. The appearance of both C and F elements in all of the above cases can be justified by the PTFE preparation, which has the formulation [(CF_2_-CF_2_)_n_], meaning it contains fluorine (F) and carbon (C) atoms [46]. The element C could also be found in the graph as the 3D-printed samples are manufactured using polymeric materials, which are organic [59].

### 3.3. Optical Metrology for the 3DP Filament Diameter

**Figure 7:**
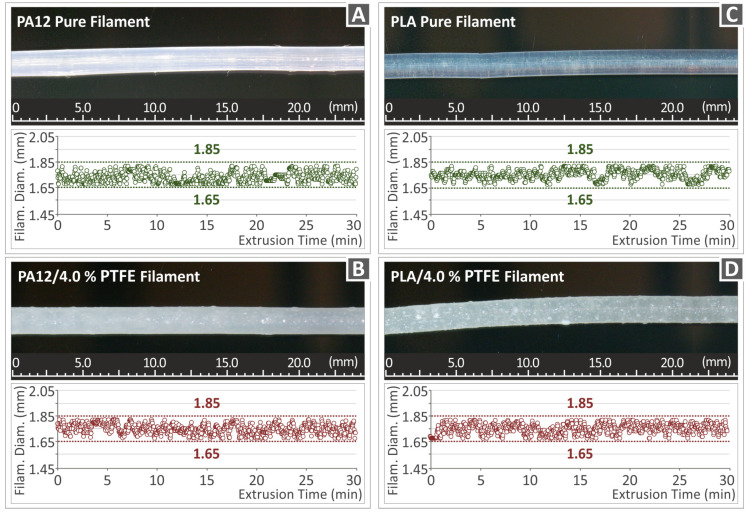
Filament diameter monitoring of (**A**) raw PA12, (**B**) neat PLA and (**C**) PA12/PTFE 4 wt.%, (**D**) PLA/PTFE 4 wt.%.

Figure 7 presents the filaments’ diameter for both neat PA12 and PLA polymer matrices and PA12/PTFE 4 wt.%, PLA/PTFE 4 wt.% loaded nanocomposites, which were all real-time recorded throughout the extrusion time. The filament diameter standard for FFF 3DP manufacturing is supposed to be around 1.75 mm. The extrusion process, as mentioned, was conducted with the utilization of the single screw extruder 3DEvo Composer 450 (from 3DEvo B.V, Utrecht, The Netherlands), which also features an integrated device responsible for measuring the filaments’ diameter throughout the whole extrusion procedure. The recorded diagrams reveal that the filament diameter remains between the span of 1.68 mm ± 0.07 mm as a result of the proper extrusion setting set and the well-functioned extruder’s capability to regulate the extrusion speed-in-line and keep a persistent diameter of the filament for the majority of its extent. In general, it is important to employ high-quality filaments in the 3D printing process in terms of including dispersion of nanoparticles, diameter homogeneity, roundness, etc., to accomplish the manufacturing of high-quality 3DP items. Considering the microscope side surface images taken from the filament presented in Figure 7, the surfaces were characterized by smoothness. Therefore, it can be assumed that the filament was of high quality, attributed to the suitable parameters utilized in the extrusion procedure.

### 3.4. Raw Polymer and Nanocompound 3DP Filament AFM Surface Roughness Analyses

**Figure 8:**
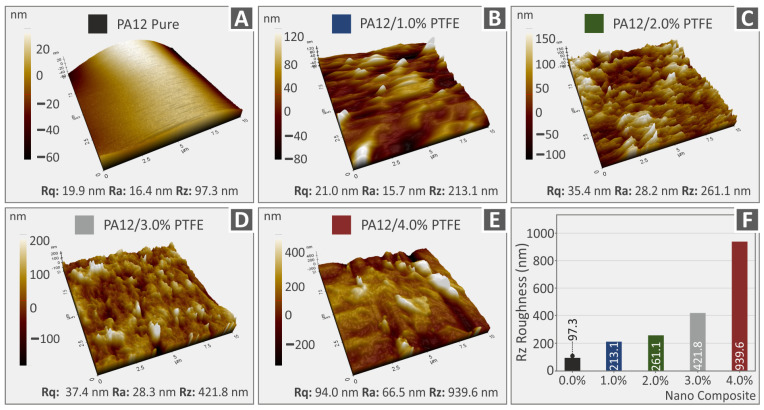
(**A**–**E**) AFM topography analysis and the Rq, Ra, Rz values, respective to PA12 pure as well as all the different filler loadings of PA12/PTFE nanocomposites, (**F**) Rz roughness spectrum.

**Figure 9:**
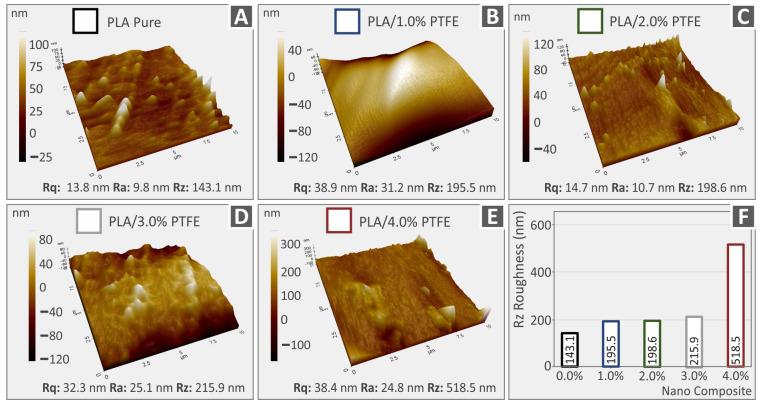
(**A**–**E**) AFM topography analysis and the Rq, Ra, Rz values, respective to PLA pure as well as all the different filler loadings of PLA/PTFE nanocomposites, (**F**) Rz roughness spectrum.

Figure 8A–E and Figure 9A–E depict 3D AFM topography images, coming from this study’s various 3DP extruded filaments, which were eventually used for the FFF 3DP manufacturing procedure in order to produce specimens for mechanical characterization. In particular, Figure 8 depicts the corresponding values of surface roughness parameters, namely Rq for root-mean-square roughness, Ra for the typical surface roughness, Rz in relation to the distinction between the highest “peak” and the deepest “valley” on the surface of pure PA12 and PA12/PTFE 1.0–4.0 wt.% along with the topography pictures. Figure 9 also shows not only the topography images but also the related Rq, Ra, and Rz roughness values for pure PLA and PLA/PTFE at 1.0 to 4.0 weight percent. It is shown that as long as the amount of PTFE added in the PA12 and PLA increases, the roughness of the filaments also increases. The observation of the surface’s roughness increase in the nanocomposites could be due to either the presence of nanoparticles in the filament surface or to the different conformation PA12 and PLA polymer chains have formed compared to the neat polymeric materials [60,61].

Figure 8F and Figure 9F summarize in a graph the corresponding Rz surface roughness values. It is depicted that, apart from PA12 in its pure form, PA12-based nanocomposite filaments have a more increased surface than the corresponding PLA ones. The differences could be an outcome of the diverse rheological values of the examined thermoplastics as it is capable of affecting the structure of the materials’ surface [62]. Each thermoplastic’s structure is affected in its way by the addition of the PTFE filler. It is also important to mention that the measurements were taken at various positions, so it is anticipated that there will be a few differences when also taking into consideration the topography of the measurements’ microscale region.

### 3.5. Tensile Characteristics of Filaments and 3DP Samples for PLA, PA12 and All PA12/PTFE and PLA/PTFE Nanocomposites

**Figure 10:**
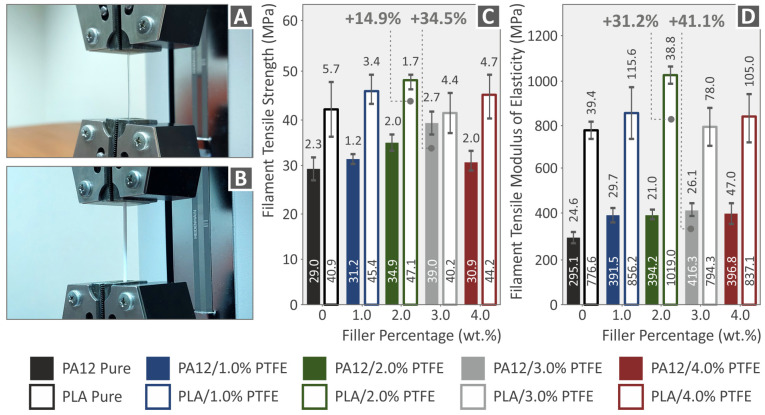
(**A**,**B**) The procedure of neat PA12 and PLA filament tensile testing, (**C**,**D**) neat PA12, PLA, and all different filler loadings of PA12/PTFE, PLA/PTFE filament tensile strength, and modulus of elasticity graphs.

The tensile tests conducted for both the PLA and PA12 nanocompound filaments are shown in Figure 10. The experimental setup for the neat polymer and the nanocomposite filaments are shown in Figure 10A,B, correspondingly. Figure 10C,D present the filaments’ tensile modulus of elasticity and tensile strength properties (standard deviation and average values) of all the pure PA12 and PLA and the nanocompounds with different PTFE concentrations produced in this research. All the filaments containing PTFE exhibit a reinforcement as per the unfilled matrices. The optimal tensile strength, according to the results, was found in the case of PLA/PTFE 2.0 wt.%, improved by 14.9% as per the pure matrix and PA12/PTFE 3.0 wt.%, improved by 34.5% as per the pure matrix. The highest modulus of elasticity was observed at PLA/PTFE 2.0 wt. %, improved by 31.2% as per the pure matrix and PA12/PTFE 3.0 wt.%%, improved by 41.1% as per the pure matrix. It can be concluded that the PLA reinforcement is significantly lower than the PA12 when it comes to strength; still, the PLA polymer becomes stiffer with the addition of PTFE.

**Figure 11:**
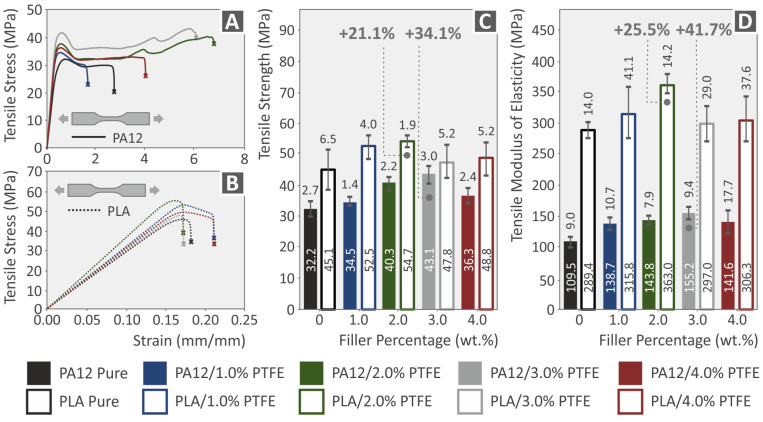
(**A**,**B**) Tensile stress/strain graphs of PA12 and PA12/PTFE nanocomposites, PLA and PLA/PTFE nanocompounds, (**C**,**D**) neat PA12, PLA and all different filler loadings of PA12/PTFE, PLA/PTFE 3DP specimens’ tensile strength and tensile modulus of elasticity graphs (standard deviation for the six samples tested in each case).

The tensile stress over strain diagrams of PA12, PLA, PA12/PTFE, and PLA/PTFE nanocomposites, calculated from the corresponding tests of 3D-printed dog-bone-shaped samples, are presented in Figure 11A,B. In Figure 11C,D the modulus of elasticity and the tensile strength properties (mean values and standard deviation are presented). According to the results, the maximum tensile strength was found in the case of PLA/PTFE wt.% 2.0, improved by 21.1% as per the pure matrix and PA12/PTFE 3 wt.% improved by 34.1% as per the pure matrix. The highest modulus of elasticity was found at PLA/PTFE wt.% 2.0, improved by 25.5% as per the pure matrix and PA12/PTFE 3 wt.%, improved by 41.7%, as per the pure matrix. A similar reinforcement effect between the two matrices (PLA and PA12) was found in this test on the 3D-printed samples.

It should be mentioned that PTFE nanocomposites performed better at the 2.0 wt.% and 3 wt.% concentration range (2 wt.% gave the highest results in the PLA nanocomposites, 3 wt.% in the PA12 nanocomposites) when it came to the extruded filaments and the 3D-printed specimens. At the highest concentration of 4 wt.%, the performance of the PA12 nanocomposites started to decline, indicating that the saturation threshold was approached at this loading. The PLA nanocomposites’ performance was rather similar to that of the 3 wt.% loading, indicating that the saturation threshold was at slightly higher concentrations than the PA12 polymer.

### 3.6. Flexural Tests of the 3DP PLA, PA12 and All PA12/PTFE, PLA/PTFE Nanocomposites

**Figure 12:**
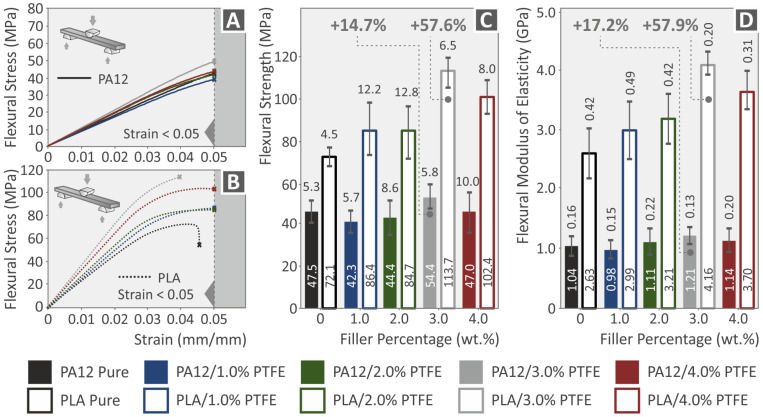
(**A**,**B**) Flexural stress/strain graphs of PA12 and PA12/PTFE nanocomposites ((**A**)—solid lines), PLA and PLA/PTFE nanocomposites ((**B**)—dotted lines), (**C**,**D**) neat PA12, PLA and all different filler loadings of PA12/PTFE, PLA/PTFE 3DP specimens’ flexural strength and modulus of elasticity graphs.

The flexural stress over strain diagrams of PA12, PLA, PA12/PTFE, and PLA/PTFE nanocomposites are presented in Figure 12A,B. The flexural modulus of elasticity and strength properties (mean values and typical deviation) of all the pure PLA and PA12 as well as their nanocomposites with PTFE are presented in Figure 12C,D. The highest flexural strength was observed at PLA/PTFE wt.% 2.0, improved by 14.7% as per the pure matrix and PA12/PTFE 3 wt.%, improved by 57.6% as per the pure matrix. The highest value of flexural modulus of elasticity was found at PLA/PTFE wt.% 3.0, improved by 57.9% as per the pure matrix, and PA12/PTFE 3.0 wt.%, improved by 17.2% as per the pure matrix. It can be observed that the PLA reinforcement reached much higher values than the PA12 one in this test. The addition of PTFE initially reduced the performance of the PA12 nanocomposites compared to the pure polymeric matrices for concentrations up to 2 wt.%. At the 4 wt.% loading, the performance in the flexural tests decreased for all nanocomposites (using PA12 and PLA as the matrix material). For this reason, it was decided not to increase further the loading even in the PLA polymer, which did not show inferior properties in the tensile tests at this loading compared to the 3 wt.% nanocomposite.

### 3.7. Mechanical Properties Results: Toughness, Impact Test, Vickers Microhardness Measurements

**Figure 13:**
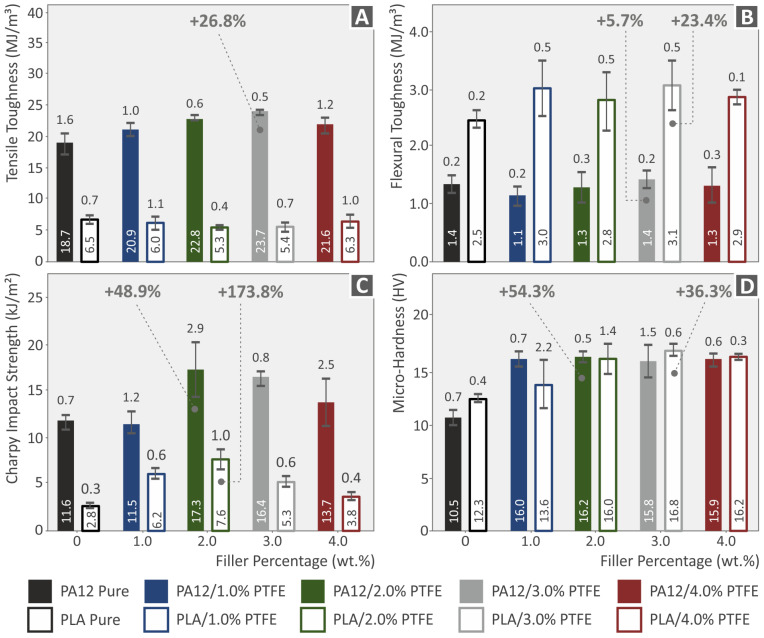
(**A**) Tensile toughness, (**B**) flexural toughness, (**C**) Charpy impact strength, (**D**) microhardness graphs showing the properties of PA12, PLA, and PA12/PTFE, PLA/PTFE nanocomposites.

Figure 13 depicts the Vickers microhardness, Charpy (notched) impact strength, and tensile and flexural hardness test results from the neat PA12, PLA, and PA12/PTFE, PLA/PTFE nanocomposites. The highest value of tensile toughness appears at PA12/PTFE 3 wt.%, improved by 26.8% as per the pure matrix; in the instance of flexural PA12/PTFE 3 wt.%, improved by 5.7% as per the pure matrix and PLA/PTFE 3.0 wt.%, improved by 23.4% as per the pure matrix. PA12 and PLA with 2.0 wt.% PTFE concentration presented the highest values on the Charpy impact strength diagram, with 48.9% and 173.8% increases achieved compared to the unfilled matrices, respectively. The highest values in the microhardness measurements were found at the PA12/PTFE 3.0 wt.% and PLA/PTFE 3 wt. % with 54.3% and 36.3% increase achieved compared to the unfilled matrices, respectively. It should be mentioned that the tensile toughness of the neat PLA was higher than all the PLA NPs.

### 3.8. PTFE Nanoparticles SEM Analysis, Lateral and Fracture Surface of 3DP Tensile Test Specimens

**Figure 14:**
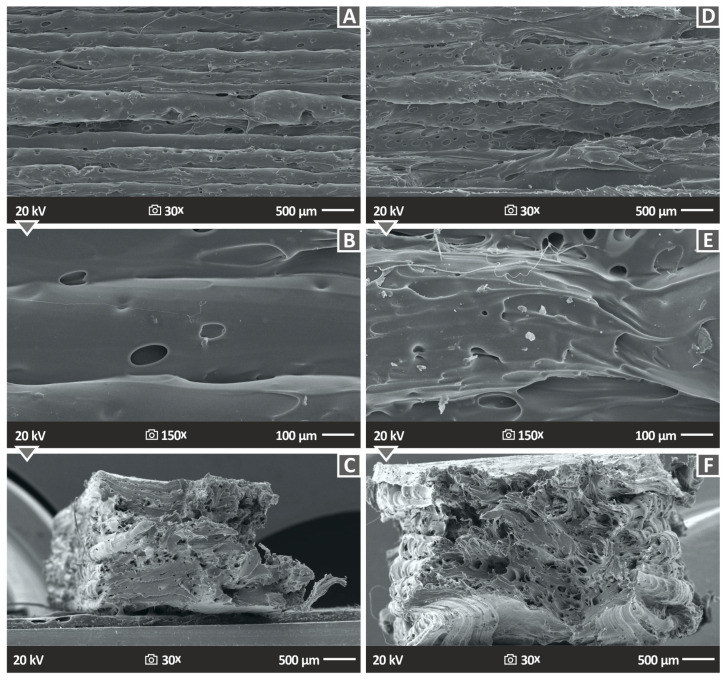
SEM analysis images of the PA12/PTFE wt.%, PA12/PTFE 4 wt.% (**A**,**B**,**D**,**E**) side surface in two different magnifications (30× and 150×) and (**C**,**F**) fracture surfaces in 30× magnification.

**Figure 15:**
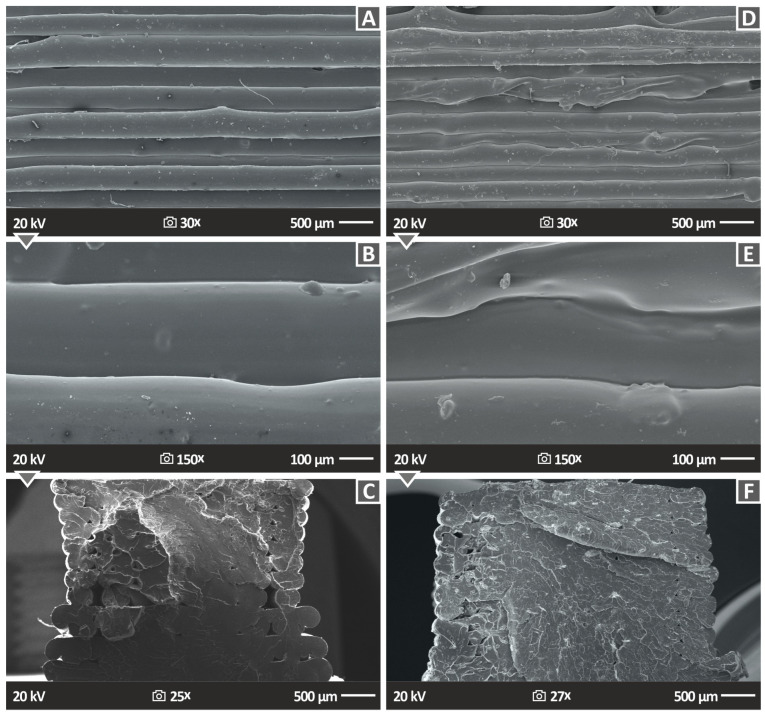
SEM analysis snapshots of the PLA/PTFE 2 wt.%, PLA/PTFE 4 wt.% (**A**,**B**,**D**,**E**) side surface in two different magnifications (25× and 150×) and (**C**,**F**) fracture surfaces in 25× and 27× magnification.

Figure 14 and Figure 15 depict the side surface and the tensile test fractured morphology of the PA12 and PLA with PTFE 2 and 4 wt.% loadings, correspondingly, exhibiting the external structure that the 3D-printed specimens resulted to have. Figure 14A,B,D,E consist of the two different magnifications (30× and 150×) of PA12 with PTFE 2.0 and 4.0 wt.% nanocomposites, while Figure 15A,B,D,E present the corresponding PLA nanocomposites images. The structure and fusion of the specimens’ layers are characterized as almost perfect in the instance of the PLA nanocomposites and as decent in the instance of the PA12 ones, allowing us to consider that the chosen 3D printing parameters gave a good-quality result. It should be stated that the 3D printing settings were optimized for the pure polymeric matrices, so such outcomes are expected. All the specimens were built with the same 3DP settings in order to be comparable. There are some pores existing on the surface mainly of the PA12 samples, while it can also be observed that the width of the layers is not the same at different spots of the specimen. There are some voids on the surface, but no discontinuities or cracks are depicted, lowering the quality of the PA12 specimens. This is in agreement with the mechanical experiments outcomes since the samples showed an improvement in their mechanical properties in comparison to both the pure polymers. The absence of extensive defects indicates an acceptable allocation of the PTFE nanofiller in the polymer matrices, which prevents the creation of micro aggregates, a factor capable of even blocking the 3D printing nozzle during the fabrication of the parts and preventing it from giving a homogenous product.

Figure 14C presents the fractured surface of PA12/PTFE 2.0 wt.%, while Figure 14F shows PA12/PTFE 4 wt.% and Figure 15C,F the corresponding PLA specimens (PLA/PTFE 2.0 wt.% and 4 wt.%) after the tensile test at 30× (Figure 14C,F), 25× (Figure 15C) and 27× (Figure 15F) magnifications. The PA12/PTFE nanoparticles show many pores as well as some discontinuities, while in the case of PLA/PTFE nanoparticles, the material seems to be well-homogenized with the existence of some pores, which is normal in 3D printing structures [63]. PA12 samples failed with a ductile failure, developing extensive neck (the cross-section of the sample at failure is significantly smaller than the nominal specimen’s section) and high deformation (Figure 15C). This is normal behavior for the PA12 polymer [64]. On the other hand, PLA fails with minimum deformation, exhibiting a rather brittle failure. These findings are in agreement with the stress–strain graphs presented above (Figure 11A,B).

**Figure 16:**
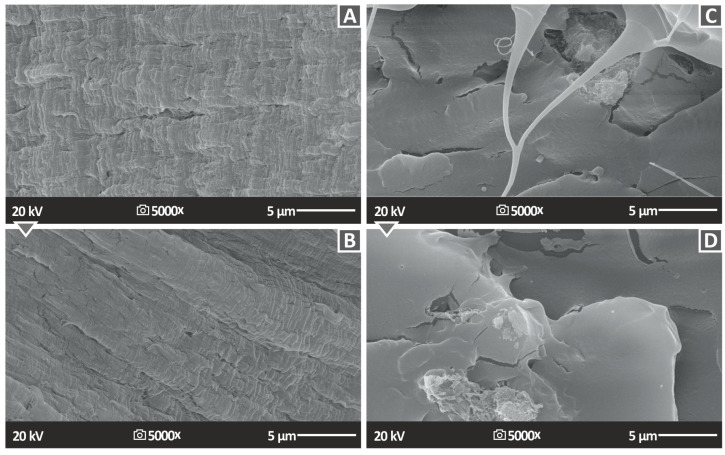
SEM analysis images of (**A**) PA12/PTFE 2 wt.%, (**B**) PA12/PTFE 4 wt.%, (**C**) PLA/PTFE 2 wt.% and (**D**) PLA/PTFE 4 wt.% specimens fractured surface at 5000× magnification.

Figure 16 shows the SEM morphological analysis results, originating from the tensile test specimen fractured surfaces, at 5000× magnification. Figure 16A presents the PA12/PTFE 2.0 wt.% and Figure 16Β presents PA12/PTFE 4.0 wt.%, where there are no pores and discontinuities; still, especially in the higher loading sample (PA12/PTFE 4.0 wt.%), the high deformations in the surface are evident. Figure 16C presents the PLA/PTFE 2.0 wt.% and Figure 16D present PLA/PTFE 4.0 wt.%. In that case, a rough, multilevel surface can be observed, confirming the brittle failure of the PLA samples. In the higher magnification images presented, microcracks are visible in all samples, which contributed to the failure of the samples. Higher magnification images were not possible to be acquired in the PLA nanocomposites, since the PLA polymer was burned. In the PA12 polymer, it was possible to acquire higher magnification images on SEM but they were not taken in order to have comparable images in the nanocomposites using the two different matrix materials.

## 4. Discussion

In this study, the authors conducted a novel investigation utilizing PTFE as a reinforcement agent in MEX 3D printing. As far as the authors are aware, this is the first time such an evaluation has been performed in the literature. The researchers focused on two commonly used polymeric matrices, namely PA12 and PLA. PA12 is a medical-grade polymer, while PLA is known for its biocompatibility. The primary objective was to incorporate PTFE, a high-performance fluoropolymer widely used in medical applications, as a reinforcement material. This objective was successfully accomplished. The resulting nanocomposites exhibited significant potential for various applications, both in their respective fields and in engineering applications. The hypothesis was proven, and PTFE managed to enhance the tensile strength of PA12 by 34.5% (with 3.0 wt.% loading) and PLA by 14.9% (with 2.0 wt.% loading). The calculated young modulus in the tensile tests is in agreement with the literature [12,48].

Therefore, the two matrices showed different reinforcement effects with the addition of PTFE, attributed probably to different interactions between the matrix and the filler. In the flexural tests, the reinforcing effect was different with an increase in the PLA nanocomposites, reaching an impressive 57.6% (with 3.0 wt.% loading), while the corresponding improvement in the PA12 nanocomposites reached 14.7% (with 3.0 wt.% loading). In both the tensile and the flexural tests, the addition of PTFE more intensively increased the stiffness of all the nanocomposites, mainly due to its characteristics, which justify such an effect. The tensile tests on the filaments produced similar results regarding the reinforcement effect with the addition of PTFE with the 3D-printed samples, which is an indication of the reliability of the reported results herein. In the impact tests and microhardness measurements, the effect of the PTFE addition was more impressive, with the increase in the impact tests reaching 48.9% for the PA12 nanocompounds, achieved with 2 wt.% loading. The corresponding increase for the PLA nanocompounds reached an impressive 173.8% (also with 2 wt.% loading). In the microhardness measurements, the increase reached 54.3% for the PA12 nanocomposites, achieved with 2.0 wt.% loading, and 36.3% for the corresponding PLA nanocomposites (with 3.0 wt.% loading). Such an impressive increase in the performance of the polymeric matrices in these tests can again be attributed to the properties of the PTFE for these types of loading. Therefore, the nanocomposites can be more suitable and recommended for applications requiring high performance in such types of loading.

It should be noted that notched samples were tested in the impact tests. The notched geometry of the impact specimens is instructed in the ASTM D6110-04 guidelines, which were followed in the study. The notch is a stress concentration feature, and the standard requires the study of the samples under these conditions. The samples’ behavior with and without the notch is expected to be the same; nevertheless, the impact strength values are expected to differ. In this study, the focus was on the WC additive effect on the mechanical properties; therefore, most of the remaining experimental parameters were not changed for comparison purposes. The literature reports that the notch does not significantly affect the response in the impact test on metal parts, while its effect on 3D-printed polymeric parts is rather important, with higher differences between the notched and un-notched specimens [65,66].

**Figure 17:**
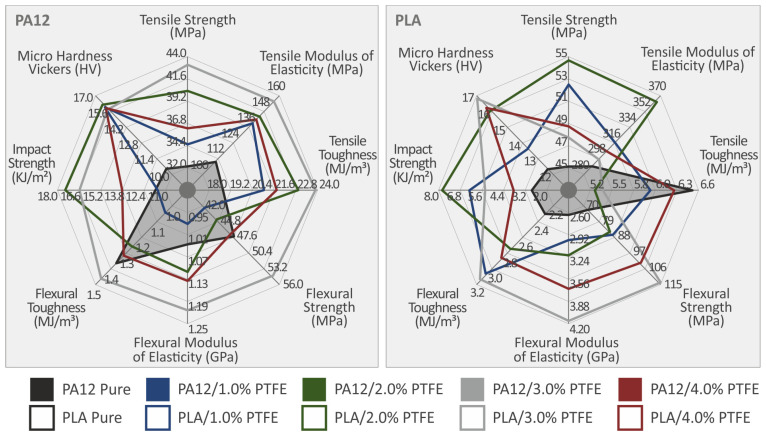
The mechanical properties of neat PA12, PLA, and 1.0, 2.0, 3.0, 4.0 wt. % PA12/PTFE and PLA/PTFE 3DP nanocomposites, resulting from all the tests conducted in this research, presented in a spider graph.

Figure 17 summarizes the data resulting from all the tests in two different spider histograms, one for the PA12 and one for the PLA nanoparticles, presenting the 3DP specimens’ mechanical properties. It is shown that the addition of PTFE nanofiller in PA12 was only beneficial for the microhardness Vickers, tensile strength, modulus of elasticity, and stiffness. The flexural toughness, strength, and modulus of elasticity improved by increasing the PTFE concentration but without getting better than the corresponding characteristics of neat PA12. On the other hand, PTFE as a filer in PLA improved most of its properties until the 2.0 wt.% or 3.0 wt.% loading. Apart from the tensile toughness, which reaches the highest (after the neat PLA) level at 4.0 wt.%, the rest of the characteristics seemed to have the highest values at 3.0 wt.% loading.

Overall, the addition of PTFE had a different effect on the two polymeric matrices, justifying the need for individual investigation of each material. Additionally, the reinforcement effect of the nanoparticles when added to a polymeric matrix differed in the different mechanical tests, also justifying the need for conducting various mechanical tests to fully characterize the prepared nanocomposites. In each test, different types of loadings (stresses) were developed in the samples. As shown by the results, the reinforcing effect was dependent on the type of stresses developed in the parts. To an extent, it can be assumed that the effect of the nanocomposites in the different types of loadings differed due to differences in the interaction between the matrix and the filler. As the filler loading increased, the increase in the reinforcing effect did not have a linear effect as expected. This nonlinear response is owed to various factors, such as the saturation of the filler in the matrix, among others [67]. It should be noted that in the impact tests, the fracture mechanism of the polymeric samples has been reported in the literature and depends on the response of the material, i.e., brittle, ductile, etc. The response is also affected by the impact test speed (strain rate) [68,69,70]. The findings of the current research are in agreement with the literature regarding the response of the polymeric matrices with the addition of fillers. The literature reports that, in several cases, the addition of fillers improves the response of the polymeric matrices under impact loading [71,72,73].

Due to the notable variations in performance observed, it is not possible to generalize these results to other polymeric matrices. Additional experiments are necessary to assess the enhancements obtained by incorporating the PTFE filler in each specific polymer. In terms of the PTFE additive, loadings were tested up to 4 wt.%. This upper limit was chosen to avoid the saturation of PTFE in the nanocomposites, as exceeding this threshold is anticipated (especially by observing the flexural test results) to have a detrimental impact on the thermomechanical properties of the nanocompounds [74,75].

The nanocomposites were fabricated using a thermomechanical process that is easily scalable for industrial production. Upon closer examination of the fracture surfaces at higher magnification, no agglomerations of the PTFE filler were detected. This observation was further confirmed by EDS mapping conducted in various regions of the surfaces. Moreover, the acceptable deviation in the mechanical tests indicated that the composition of the nanocompounds was consistent in both matrices and across all evaluated loadings. These findings suggest that the distribution of the nanoparticles within the matrix was well-formed in the prepared samples.

Importantly, the addition of PTFE did not have any adverse effects on the thermal stability of the polymeric matrices. This positive outcome ensured that the prepared nanocomposites remained stable under thermal loading conditions. Additionally, the TGA demonstrated that the nanocomposites started to degrade at higher temperatures compared to the processing temperatures employed in the current study. This result signifies the robustness of the fabrication process and confirms that the acquired results were not influenced by such degradation phenomena.

The results presented In this study cannot be directly compared to the existing literature due to the absence of similar nanocomposites for MEX 3D printing that has been prepared using the proposed methodology. As the literature review section indicates, research on PTFE in MEX 3D printing is still limited. Therefore, the outcomes of this report provide novel insights and contribute to the existing knowledge gap in the field of nanocomposites for MEX 3D printing. Additives in NPs forms, such as silver NPs [48] prepared in a similar way for MEX 3D printing applications, achieved higher reinforcement effects on the tensile and flexural tests for the two polymeric matrices but lower on the impact and microhardness measurements, indicating that each additive is recommended for specific types of applications. Comparing the effect of carbon and glass fillers in the two polymeric matrices (PA12 and PLA) to the results presented herein, it was found that carbon [76] and glass fibers [77] improved the polyamide matrix mechanical response in a better way than the WC NPs. Still, the addition of fibers that are not on the nanoscale had a different reinforcement mechanism on the polymeric matrices, and the preparation process also differed. In another work with PA12, the carbon fibers and glass bubble reinforcement effect was similar to what was achieved and reported herein with the addition of the WC NPs [13]. For the PLA polymer, the addition of carbon fillers showed an inferior reinforcement effect, compacted to the current study results [78,79].

Regarding the cost of improving the mechanical strength of the two polymeric matrices, a rough estimation of laboratory scale costs showed that the process can be considered as cost-effective. Considering that the cost ratio between the raw material and the commercial filament is 1/10 (PLA raw material costs about EUR 1.8/Kg, that is EUR 0.0018/g, while 1 Kg PLA filament costs about EUR 20), it can be safely assumed that main cost is related to the filament preparation (extrusion process) and the process until it reaches the market. The PA12 medical grade studied costs about EUR 0.013/g, while the PTFE costs about EUR 0.254/g. All these prices are for laboratory-scale use and are significantly lower for industrial-scale use. With the methodology, the additional costs compared to the unfilled polymer filament are the following cost of the additive and the cost of the mixing process, which can be safely considered insignificant. The addition of 3 wt.% PTFE to the PA12 polymer, which had the optimum mechanical performance, has an additional cost in the raw materials of EUR 0.254/g × 0.03, which is EUR 0.00762/g, so the cost per gram increases from EUR 0.013/g to EUR 0.02062/g. For the PLA polymer, 2 wt.% PTFE had the optimum mechanical performance, which adds a EUR 0.254/g × 0.02 (EUR 0.00508/g) cost in the raw materials, so the cost, in this case, increases from EUR 0.0018/g to EUR 0.00688/g. For both matrices, the increase can be considered normal since the cost of the raw materials has a low contribution to the overall cost, as mentioned.

## 5. Conclusions

This work presented the results arising from the production of PA12 and PLA nanocomposite 3D-printed filaments using the procedure of melt-mixing and combining, with the ultimate aim of improving the thermomechanical performance of 3D-printed specimens. The results coming from those processes were positive, as they showed an upgrade in the mechanical behavior in the majority of the nanocomposite concentrations. As reported, there were observed differences in the response between the two polymers (PA12 and PLA) in this study.

The PTFE filler was used in 1, 2, 3, and 4 wt.% loadings in both the PLA and PA12 polymeric matrices. The goal of this investigation was to examine how the inclusion of PTFE nanocompounds affects the thermomechanical properties of 3D-printed nanocomposites. Overall, the 3 wt.% loading for the PA12 polymer and the 2 wt.% loading for the PLA polymer were the optimum WC filler concentrations for each matrix, respectively. In the higher loading studied (4 wt.%), the mechanical properties started to decrease for both polymeric matrices, indicating that saturation of the filler in the matrix started to occur, which negatively affected the mechanical performance of the nanocomposites [80]. So, increasing the WC concentration in the nanocomposites has no positive effect on their mechanical performance beyond a specific loading. The exact saturation threshold was not determined, as it was not within the scope of the work.

These nanocompounds hold potential for various engineering applications, particularly those requiring high impact and hardness strength. To assess this, 3D-printed prototype specimens were created using the produced filaments according to various ASTM protocols. Subsequently, mechanical tests such as tensile, flexural, impact, and microhardness evaluations were conducted. Additionally, SEM analysis was performed on the lateral surface morphology and the cracked surfaces of the tensile samples to assess the influence of different nanofiller loadings on the 3D-printed samples.

Exclusively, this study is dedicated to inspecting the impact of PTFE nanoparticles on the mechanical characteristics of polylactic acid and polyamide 12 in the context of 3D fused filament fabrication (FFF) printing. The research successfully determined how the inclusion of PTFE nanocompounds affected the mechanical responses of these materials. In the future, further exploration will be carried out to investigate other properties such as wear resistance and rheology of these materials. The results of this study indicate the potential of PTFE nanocompounds as fillers for the creation of multifunctional nanocompounds in MEX 3D printing.

## Figures and Tables

**Table 1:** Extrusion settings per nanocompound in the 3DEvo composer 450 (Utrecht, The Netherlands). The screw and the winder speed were the same in all nanocompounds.

	Heat Zone 1 (Near the Nozzle) (°C)	Heat Zone 2 (Central Zone) (°C)	Heat Zone 3 (Central Zone) (°C)	Heat Zone 4 (Near the Hopper) (°C)
PA12, PA12/PTFE	210	220	220	185
PLA, PLA/PTFE	195	205	205	175
	Screw speed:	8.5 rpm	Winder speed:	3–15 rpm

**Table 2:** Identification of the main Raman peaks of PA12 pure and their related assignments.

Wavenumber (cm^−1^)	Raman Peak Assignment
1000	C-H in-plane bending [49]
1029	C-C and C-O vibration [50]
1062	C-O-C stretching [49]
1106	C-O-C stretching [49]
1153	Skeletal deformation [50]
1193	C-O-C stretching [51]
1294	C-O-C stretching [49]
1435	CH_2_ deformation [49,52]
1600	Skeletal vibration of the C=C aromatic ring [53,54]
1632	v (C=C) [55]
2849	CH_2_ symmetric stretching [50]
2883	CH_2_ symmetric stretching [50,56]
2899	CH stretching [49,56]
2922	CH_2_ asymmetric stretching [50]

**Table 3:** Identification of the main Raman peaks of PLA pure and their related assignments.

Wavenumber (cm^−1^)	Intensity	Raman Peak Assignment
870	Medium	C-COO stretching [57]
1040	Small	C-CH_3_ stretching [57]
1059	Small	C-C asymmetric stretching
1126	Medium	C-O-C stretch [51]
1293	Medium	C-O-C stretch [52]; C-H_2_ twisting [52]
1413	Small	C-H_3_ deformation [49]
1437	Medium	C-H_3_ deformation [49] C-H_2_ deformation [52]
1457	Medium	C-H_3_ symmetric bending [49,51,57]; C-H_2_ twisting [52]
1770	Medium	C=O stretching [51,57]
2721	Small	C=O stretching [58]
2845	Major	C-H_2_ symmetric stretching [50]
2880	Major	C-H_2_ symmetric stretching [50]; C-H symmetric stretching [56]
2945	Major	C-H_2_ asymmetric stretching [50]
3000	Medium	C-H_3_ asymmetric stretch [56]

## Data Availability

The data presented in this study are available upon request from the corresponding author.
